# Influence of repeated cycles of full-strength sodium hypochlorite activation on the mechanical behavior of root canal-treated teeth: a laboratory study

**DOI:** 10.1186/s12903-026-08082-3

**Published:** 2026-04-01

**Authors:** Mohammed Hammad Abdu Ismail, Ahmed Mohamed Bakry, Mohammed Turky

**Affiliations:** 1 Department of Endodontics, Faculty of Dentistry, Minia University, Minia, Egypt; 2 Department of Maxillofacial Radiology, Faculty of Dentistry, Minia University, Minia, Egypt; 3 Department of Endodontics, Faculty of Dentistry, Sphinx University, Assiut, Egypt

**Keywords:** Endodontics, Root canal therapy, Sodium hypochlorite, Tooth fractures, Ultrasonic therapy

## Abstract

**Objectives:**

To assess the effects of different activation protocols of full-strength sodium hypochlorite (NaOCl), including stepwise intraoperative activation (SIA), conventional ultrasonic irrigation (CUI), and their combination (SIA + CUI), compared to conventional syringe irrigation (CSI), on the fracture resistance of root canal-treated teeth.

**Methods:**

Ninety extracted maxillary second premolars were assigned to four experimental groups (*n* = 15 each) based on the irrigation protocol: CSI, SIA, CUI, and SIA + CUI, along with two control groups (*n* = 15): intact teeth (positive control) and saline irrigation (negative control). Standardized access cavities were prepared, followed by uniform root canal instrumentation, while irrigation protocols were conducted as assigned. Root canals were filled, and access cavities were sealed with resin composite. Specimens underwent compressive fracture testing using a universal testing machine to determine the maximum load to fracture after thermo-mechanical fatigue, and failure modes were assessed. Data were statistically analyzed, with the significance threshold set at *p* < 0.05 and a 95% confidence interval (CI).

**Results:**

All experimental groups, along with the negative control group, exhibited significantly reduced fracture resistance and notable irreparable failure patterns in comparison to intact teeth (563.92 ± 28.52 N; CI: 548.13–579.71) (*p* < 0.001). No statistically significant differences in the maximum load to fracture were observed among the CSI (473.90 ± 13.94 N; CI: 466.18–481.62), CUI (470.94 ± 12.5 N; CI: 464.02–477.87), SIA (468.15 ± 9.68 N; CI: 462.79–473.51), SIA + CUI (467.61 ± 6.74 N; CI: 463.88–471.35), and the negative control group (474.45 ± 5.62 N; CI: 471.34–477.56), despite increased volumes and longer exposure to NaOCl in activation groups (*p* > 0.05). The majority of failure modes were catastrophic across the tested and NC groups, though distribution did not vary significantly (*p* = 0.06).

**Conclusion:**

The repeated activation of full-strength NaOCl via SIA, CUI, or their combination did not compromise fracture resistance compared to CSI. Root canal treatment itself reduced structural integrity relative to intact teeth, with a noted increase in the likelihood of unfavorable fracture types, underscoring the necessity for meticulous preservation of tooth structure during root canal procedures.

**Supplementary Information:**

The online version contains supplementary material available at 10.1186/s12903-026-08082-3.

## Introduction

Root canal irrigation constitutes a fundamental component of successful root canal treatment, serving a vital function in accessing hard-to-reach areas within the root canal system to disrupt microbial biofilms, eliminate remnants of pulp tissue, and remove residual debris. This process is essential in complementing the mechanical action of shaping files. Among the variety of irrigation solutions available, NaOCl is widely regarded as the gold standard due to its strong antimicrobial properties and tissue-dissolving capabilities. There is no universally established consensus regarding the ideal concentration of NaOCl for clinical settings, given its efficacy across a spectrum of concentrations. However, many practitioners gravitate towards utilizing its full-strength formulation to leverage its maximum effectiveness [1, 2].

Nonetheless, employing NaOCl even in full-strength form through the conventional irrigation method, known as the CSI, raises concerns regarding the thorough disinfection and cleaning of the entire root canal system [2]. This method relies on the positive pressure generated by the fluid as it is expelled through the needle, in conjunction with the viscosity of the irrigant, to facilitate flow throughout the root canal [3]. However, studies have indicated that the effectiveness of CSI is typically limited to a distance of about 1 mm from the needle tip, leaving regions further away inadequately cleaned and disinfected [4]. This limitation can adversely affect treatment outcomes, as insufficient disinfection may lead to persistent infections [4]. Moreover, the closed-ended nature of the root canal system contributes to the potential accumulation of air bubbles in the apical region, resulting in a phenomenon known as “apical vapor lock.” [5]. This situation can significantly hinder the penetration of irrigating solutions into the critical apical section of the root canal, which poses the most challenging territory for cleaning and disinfection [5]. Additionally, the inherent viscosity of NaOCl can limit the flow of irrigants to these crucial areas.

To mitigate these challenges, various strategies have been developed to enhance the flow and efficacy of endodontic irrigants. One promising approach involves activating the irrigating solutions at the end of root canal preparation, which employs mechanical, physical, or other forms of energy to enhance their distribution throughout the intricate root canal architecture [3]. Among the array of techniques utilized for irrigant activation, passive ultrasonic irrigation (PUI) has emerged as the most frequently employed activation method. A recent systematic review has indicated the effectiveness of PUI when compared to the other activation approaches regarding root canal disinfection—a critical component of predictable root canal treatment [6]. Research has highlighted that ultrasonic activation generates acoustic streaming and cavitation, resulting in increased energy transfer within the irrigant solutions [7, 8]. This energy facilitates a greater shear stress against the canal walls, effectively disrupting adherent pulp remnants, debris, and residual bacterial biofilms while simultaneously alleviating the issue of apical vapor lock [7, 8].

Furthermore, ultrasonic activation has been found to elevate the temperature of the irrigants, which decreases their viscosity and enhances their flow characteristics within the canal system [9]. This increase in thermal energy not only promotes better penetration of the irrigants but also enhances their chemical reactivity, thereby bolstering their antimicrobial and tissue-dissolving capabilities [9]. The significant role of ultrasonic activation in disinfecting root canals has been documented in various studies, including a recent systematic review and meta-analysis of randomized clinical trials that has demonstrated its superiority over CSI in achieving more effective root canal disinfection [10]. However, it is important to note that PUI does not completely eradicate bacteria from root canals, which may compromise treatment success [11]. Consequently, additional disinfection and debridement protocols are advisable to attain optimal results.

In recent discussions, SIA has attracted attention for its potential to improve the efficiency of root canal debridement and disinfection [12–14]. SIA involves the activation of additional fresh NaOCl solution after each mechanical instrument and CSI during root canal preparation, providing larger volumes and extended exposure time to NaOCl. While existing research has proven the antimicrobial efficacy and cleaning effectiveness of this approach [12–14], its specific impact—both independently and in conjunction with conventional activation methods—on the mechanical integrity and fracture resistance of root canal-treated teeth remains relatively unexplored. Given that mechanical properties are crucial for the long-term retention of root canal-treated teeth, this in vitro study aimed to evaluate the influence of activating the full-strength NaOCl using CUI and SIA, both alone and in combination, on the fracture resistance of these teeth, compared to the non-activation conventional irrigation method (CSI).

The hypothesis being tested in the present study posits that repeated activation of the full-strength NaOCl using both SIA and the combination of SIA with conventional activation techniques leads to prolonged contact between the irrigant and dentin, which subsequently reduces the fracture resistance of root canal-treated teeth compared to CSI.

The proposed methodology aimed to fill a critical gap in the existing body of literature by systematically investigating the cumulative mechanical effects of multiple intraoperative activations of full-strength NaOCl. Unlike previous studies [15–17] that have predominantly focused on the impact of single or final activation protocols—which represent the standard practice in endodontics—this research took a more nuanced approach by examining how repeated exposures to this potent chemical agent influence the fracture resistance of root canal-treated teeth. Understanding the mechanical ramifications of repeated NaOCl activation is essential for informing clinical procedures and developing best practices that prioritize patient safety and treatment effectiveness. Ultimately, this research sought to enhance the knowledge base surrounding root canal therapy, thereby guiding clinicians in making more informed decisions regarding the use of full-strength NaOCl in their treatment protocols.

## Materials and methods

This manuscript adhered rigorously to the Preferred Reporting Items for Laboratory Studies in Endodontology (PRILE 2021) guidelines [18], which establish a robust framework for transparency, consistency, and rigor in reporting laboratory investigations in endodontics. The findings of this laboratory study are succinctly encapsulated in the PRILE 2021 flowchart, presented as Supplementary Fig. 1. This flowchart visually delineates the critical components of the study, including the primary objective, the null hypothesis formulated at the outset, the data collection and analysis methodologies employed, and a summary of the results attained. Moreover, it systematically outlines the conclusions derived from the data, facilitating a deeper understanding of the research process and its outcomes. By explicitly elucidating these elements, the flowchart serves as an essential reference for readers to grasp the key facets of the study and its broader implications within the field of endodontic research.

### Ethical considerations

The current investigation was conducted in strict accordance with the ethical standards set forth by the Declaration of Helsinki, which underscores the imperative of safeguarding participant rights and welfare in medical research. Before the commencement of the study, formal ethical approval was obtained from the Research Ethics Committee at the Faculty of Dentistry, Minia University, Minia, Egypt, under Registration No. 1136. This approval entailed a detailed appraisal of our research protocol to ensure adherence to all pertinent guidelines and regulations governing research involving human subjects.

Moreover, this study emphasized transparency and voluntary participation by securing written informed consent from all participants. The consent process involved providing prospective subjects with comprehensive details about the study’s aims, methodologies, potential risks, and benefits, thereby enabling them to make informed choices regarding their participation. Throughout the study, we meticulously adhered to ethical practices to maintain the highest standards and uphold the dignity of all participants involved.

### Sample size calculation

The sample size for the present study was calculated using G*Power software, version 3.1.9.7 (Heinrich Heine University, Düsseldorf, Germany). To determine the appropriate sample size, a one-way analysis of variance (ANOVA) test was chosen, specifically the fixed effects, omnibus, one-way model. This decision was informed by preliminary data collected from a pilot study evaluating the fracture resistance and failure modes, which included five teeth per group.

In the power analysis, an effect size (f) of 0.5 and a 95% CI were utilized. Additionally, a significance level (α) of 0.05 was set, indicating that we aimed for a 5% risk of concluding that a difference exists when there is none. Furthermore, a power (1 – β) of 0.95 was employed, suggesting a 95% probability of correctly detecting a true effect if it exists.

Based on these parameters, the analysis revealed that a total sample size of 90 teeth (15 per group) would be necessary to reliably identify a statistically significant difference among the different experimental groups being tested. This sample size ensures that the study is adequately powered to draw meaningful conclusions regarding the fracture resistance and failure patterns.

## Sample selection

A rigorous approach was employed for sample selection to ensure the validity of the findings. From an initial pool of 113 teeth, 90 freshly extracted mature sound human maxillary second premolars were selected. These were sourced from healthy patients aged 20–40 at the outpatient clinic of the Faculty of Dentistry, Minia University, Minia, Egypt. Teeth were extracted with minimal trauma for either periodontal or orthodontic reasons.

Establishing a reliable baseline is vital in research design due to the potential effects of anatomical variations on outcomes. To mitigate this risk, careful attention was given to anatomical specifics during sample selection. Three-dimensional (3D) imaging using cone-beam computed tomography (CBCT) (Papaya 3D Plus, Genoray, Gyeonggi-do, Korea) was utilized to achieve anatomical congruence in tooth morphology, dimensions, pulp space anatomy, and volume. The CBCT images were acquired with a limited field of view (FOV), measuring approximately 5 × 5 cm. This compact FOV was chosen to enhance image resolution while focusing on specific anatomical structures. The voxel size was set within a range of 0.125 to 0.2 mm, allowing for a high level of detail in the images, which was crucial for accurate assessment of root canal anatomy.

The imaging process adhered strictly to the standard exposure settings recommended by the manufacturer, ensuring optimal performance for high-resolution imaging. Following the acquisition of the images, they were reconstructed in three anatomical planes: axial, sagittal, and coronal. This multiplanar reconstruction facilitated a comprehensive evaluation of canal anatomy. The analysis was performed using software provided by the manufacturer, which allowed for precise assessment, targeting teeth with oval root canal configurations.

All selected teeth had single roots with fully developed apices, and their root canals conformed to Vertucci’s Type I canal classification [19]. These root canals exhibited an oval cross-section, with the buccolingual dimension being twice that of the mesiodistal dimension. The roots and root canals were straight. To ensure comparable working lengths, teeth wherein the canal terminus was precisely aligned with the root apex were chosen. By systematically standardizing these anatomical features, the investigation minimized variability during root canal procedures, including instrumentation and filling, and subsequent coronal restorations, hence allowing for a more precise examination of the effects stemming from varying irrigation protocols.

In addition, dimensions of the selected teeth were precisely measured using a digital caliper with an accuracy of 0.01 mm. The buccolingual dimension averaged 10.5 ± 0.5 mm, while the mesiodistal dimension averaged 8 ± 0.5 mm. To ensure consistency in tooth length across all specimens, a systematic occlusal reduction was conducted prior to performing root canal procedures. Using the digital caliper, the initial crown–root length of each tooth was meticulously measured, extending from the tip of the buccal cusp to the apex. In cases where the measured lengths exceeded the predetermined length of 20 mm, a coronal reduction was performed. The occlusal reduction process utilized a water-cooled, high-speed handpiece (NSK Pana-Max Plus; NSK Nakanishi, Tochigi, Japan) fitted with a flat-end diamond bur (ISO 111 − 012, Mani, Tochigi, Japan). This bur was instrumental in removing enamel and dentin in a manner that was perpendicular to the long axis of the tooth, ensuring a uniform reduction. The buccal cusp was specifically chosen as the reference point for this procedure, given its anatomical consistency and reproducibility across different specimens, which was critical for achieving accurate and reliable results. After completing the occlusal reduction, the final length of each tooth was re-evaluated using the digital caliper. Any specimens that deviated more than ± 0.1 mm from the target length were excluded from further analysis to maintain the integrity of the study. This rigorous protocol not only ensured that all specimens adhered to uniform dimensions but also minimized the potential impact of variations in crown height on the outcomes of subsequent mechanical testing.

To further control for variables, all specimens were restored to a standardized occlusal anatomy prior to mechanical testing. This step was crucial in eliminating any confounding effects related to cusp morphology, thereby enabling a more accurate assessment of the mechanical properties under evaluation. In this way, the experimental design was fortified to yield reliable and reproducible data.

Exclusion criteria were stringent, discarding teeth with caries (coronal or radicular), coronal restorations, prior root canal treatments, open apices, pulp calcifications (isolated or diffuse), and internal or external root resorption. These were duly replaced with samples conforming to the inclusion criteria. Critical analysis under a dental operating microscope (DOM) at 20× magnification (Magna Labomed, Labo America Inc., Fremont, CA, USA) was performed to identify any cracks or fractures.

Prior to experimentation, the selected samples underwent ultrasonic scaling to eliminate any attached hard or soft debris, followed by disinfection in a 2.5% NaOCl solution (El Nasr Company for Intermediate Chemicals, Giza, Egypt) for 30 min. The specimens were subsequently stored in a 0.1% thymol suspension (Formula e Acao, São Paulo, SP, Brazil) at 4 °C for up to one month, adhering to the technical specifications outlined in ISO/TS 11–405 established in the domain of dental materials in 2003.

### Simulation of periodontal support

In an attempt to replicate periodontal tissues and bone support, two meticulously crafted half-split Teflon molds, each measuring 25 mm in height and 30 mm in diameter, were utilized. These molds served as a precise framework for the application of self-curing resin.

To ensure a realistic simulation of periodontal anatomy, each tooth was carefully encased in a stretch film with a thickness of 0.3 mm, which served to protect the root surfaces during the resin application process. The tooth was then submerged in acrylic resin, achieving a level that extended approximately 2 mm apical to the cementoenamel junction (CEJ). This specific placement was integral in accurately replicating the biological bone level and maintaining the relationship between the tooth and its supporting structures.

A Ney Dental Surveyor (Anaheim, CA, USA) was employed to ensure that each tooth was positioned with precision within the acrylic block. This apparatus facilitated optimal centering and maintained a parallel alignment with the long axis of the tooth, critical for the simulation’s accuracy.

Once the acrylic resin had thoroughly cured and set, the teeth, still protected by the stretch film, were carefully extracted from the molds. Following this, a light-body silicone (Elite HD; Zhermack SpA, Badia Polesine, Italy) was injected into the root cavity, effectively replicating the periodontal ligament—a crucial element in the overall simulation of the periodontal environment.

To finalize the preparation, any excess impression material was meticulously trimmed away using a #12 scalpel (Swann Morton, Sheffield, England). This step ensured that the simulation maintained precision and did not compromise the structural integrity or functional aspects of the model.

### Root canal procedures

Given its impact on the effectiveness of subsequent root canal procedures, including root canal irrigation, standardized conventional access cavity designs were prepared. After marking the access center at a midpoint between the buccolingual and mesiodistal axes, the pulp chamber was initially penetrated, and its roof was completely removed using a diamond round bur # 2 (Brasseler USA, Savannah, GA, USA). Subsequently, the axial walls were refined using the Endo Z bur (Dentsply-Maillefer, Ballaigues, Switzerland) to provide smooth, slightly divergent walls to facilitate adequate visibility and accessibility. All burs were mounted on a high-speed handpiece (NSK Pana-Max Plus; NSK Nakanishi, Tochigi, Japan) with a water spray. The dimensions of access cavities were stringently standardized in all samples by means of a digital caliper with an accuracy of 0.01 mm, as follows: the buccolingual dimension was 4.5 ± 0.2 mm, the mesiodistal dimension was 3 ± 0.2 mm, and the average access depth was 6 mm from a fixed occlusal reference point.

After preparing the access cavities, the patency of the root canals was evaluated with a stainless-steel K-file, ISO size 10 (Dentsply, Maillefer, Ballaigues, Switzerland). Canals that were non-patent or had initial apical diameters exceeding 0.20 mm were excluded from the study and replaced with alternative specimens adhering to the inclusion criteria. The working length was established by introducing a stainless-steel K-file (ISO size 10) until it reached the apex, followed by a subtractive adjustment of 1 mm to account for the length measurements. Following this, a manual glide path was established utilizing stainless-steel K-files, ISO sizes 15 and 20 (Dentsply, Maillefer, Ballaigues, Switzerland).

Teeth were then equally assigned to the different study groups using a stratification process. Each set of 6 teeth with comparable ages, dimensions, and anatomical characteristics was allocated to the various study groups, including positive and negative control groups (*n* = 15), along with four experimental groups (*n* = 15) based on the irrigation protocol as follows:

- Positive Control (PC) group – Intact teeth

The present study delved into the comparison of various irrigation methods and their impact on the fracture resistance of root canal-treated teeth. In this context, the CSI method serves as the standard benchmark for comparison. To enhance the accuracy of the findings, intact teeth were utilized as a PC group (*n* = 15), allowing for a clear assessment of how different irrigation protocols influenced the mechanical behavior of the teeth following the root canal procedures. By examining the changes in fracture resistance, the study aimed to provide valuable insights into the effect of each irrigation technique.

- Experimental group - Conventional Syringe Irrigation (CSI) Group

In this cohort (*n* = 15), root canal instrumentation was achieved using Hyflex CM rotary file systems (Coltene Whaledent, Cuyahoga Falls, OH, USA), adhering strictly to the manufacturer’s specifications. This multi-file rotary system was operated in continuous rotation mode using a controlled-torque electrical endodontic motor (TriAuto mini; J. Morita MFG, CORP, Japan). For each canal, a single set of files was used. After setting the appropriate speed and torque per the manufacturer’s guidelines, coronal flaring was implemented with a 25/0.08 file, succeeded by 20/0.04 and 25/0.04 files until the predetermined working length was reached. The middle third of the canal was then enlarged using a 20/0.06 file, followed by the application of 30/0.04 and 40/0.04 instruments, up to the full working length.

Simulating the clinical scenario in optimizing the debridement and disinfection of the oval root canals, where manual instrumentation is recommended in conjunction with the engine-driven root canal preparations, circumferential filing of both buccal and lingual aspects of the root canals was executed using a stainless-steel H-file (ISO size 40) (Dentsply, Maillefer, Ballaigues, Switzerland) with three strokes per surface [20]. A 2 mL irrigation with 5.25% NaOCl was administered for 20 s between mechanical file uses, culminating in a total volume of 15 mL over 150 s. The final irrigation protocol consisted of 10 mL of 5.25% NaOCl for 2 min, followed by 10 mL of 17% ethylenediaminetetraacetic acid (EDTA) for another 2 min (Prevest DenPro Limited, Jammu & Kashmir, India), interspersed with saline rinses of the same volume for 2 min each. A final saline flush was performed to eliminate residues of the reagents used. Irrigating solutions were delivered through a 30-gauge side-vented closed-end irrigating needle (Fanta Dental Materials Co., Ltd., Shanghai, China), positioned 1 mm short of the working length in a back-and-forth motion.

- Experimental group - Stepwise Intraoperative Activation (SIA) Group

For this subset (*n* = 15), the chemo-mechanical protocols established in the CSI group were replicated, augmented by ultrasonic activation of an additional 2 mL of 5.25% fresh NaOCl for 20 s following irrigation between files. An E1 Irrisonic ultrasonic tip (Helse Dental Technology, São Paulo, SP, Brazil), equivalent to size 20/0.01, was employed and set on an ultrasonic unit (Satelec/Aceton P5 Newtron, Merignac, France) adjusted on the Endodontic Precision Mode with a frequency of 28 kHz and a power setting of 2 (20%). This tip was passively inserted to the level reached by the preceding mechanical file and irrigating needle. This enhancement resulted in a cumulative volume of 30 mL of NaOCl over 5 min, with approximately 7 cycles of NaOCl activation, during the instrumentation procedure.

- Experimental group - Conventional Ultrasonic Irrigation (CUI) Group

This group represented the standard activation method. In this cohort (*n* = 15), the chemo-mechanical preparation described in the CSI group was also followed. Additionally, at the conclusion of the chemo-mechanical preparation, passive intermittent ultrasonic activation of a fresh 2 mL of 5.25% NaOCl was conducted in three successive cycles of 20 s each, using the same ultrasonic tip and power settings as in the SIA group. The tip was inserted up to 3 mm short of the working length in root canals filled with NaOCl as a part of the final irrigation protocol, following the use of 10 mL of 5.25% NaOCl for 2 min and before EDTA application. This approach contributed an extra 6 mL of NaOCl compared to the CSI group, with 3 activation cycles occurring intermittently over a total duration of 1 min.

- Experimental group - SIA + CUI Group

In this group (*n* = 15), the root canal procedures were conducted following the CSI methodology, enhanced through the integration of both SIA and CUI strategies. Identical instrumentation and irrigation protocols were employed, amounting to a total of 15 mL of 5.25% NaOCl administered over 150 s during the instrumentation phase. The final rinse comprised a 10 mL application of 5.25% NaOCl followed by 10 mL of 17% EDTA, each for 2 min, supplemented by intermediate and final saline flushes with equivalent volumes and durations. Additionally, SIA was incorporated, utilizing 2 mL of 5.25% NaOCl for 20 s between mechanical files, which led to an extra 15 mL of NaOCl delivered over 150 s. CUI was also included, providing agitation of an additional 6 mL of 5.25% NaOCl for 60 s, resulting in approximately 10 cycles of NaOCl activation.

- Negative Control (NC) group – Saline Irrigation

This group (*n* = 15) consisted of teeth that underwent root canal and restorative procedures performed similarly to those previously described, with the sole irrigation agent being saline solution. The inclusion of this NC group was crucial for isolating and critically evaluating the effects of various irrigation protocols on the fracture resistance of root canal-treated teeth.

After completing the chemo-mechanical preparation, the root canals were thoroughly dried using matched paper points (Coltene Whaledent, Cuyahoga Falls, OH, USA). The process of drying the root canals using absorbent paper points can effectively reduce the presence of excess free fluid; however, it does not achieve complete desiccation of the dentin structure. It is essential to recognize that the intrinsic moisture contained within the dentinal tubules remains sufficiently abundant to facilitate the hydration reaction required by hydraulic calcium silicate–based sealers. These sealers possess unique properties that enable them to set and develop essential physicochemical characteristics, even in clinically dry conditions—conditions that can be adequately attained through the use of paper points. Importantly, the drying protocol employed in the present study was carefully designed to ensure that it did not compromise the hydration or overall performance of the sealers. By providing an optimal level of moisture, the integrity and functionality of the hydraulic sealers were maintained, thus allowing them to achieve their intended efficacy in the sealing of the root canal system. This understanding reinforces the notion that a strategic approach to canal drying can support the successful implementation of hydraulic sealing materials without diminishing their potential benefits.

Following canal dryness, a single-cone technique was employed to fill the root canals, utilizing matched master gutta-percha cones (Coltene Whaledent, Cuyahoga Falls, OH, USA) in conjunction with a bioceramic sealer (EndoSequence BC sealer; Brasseler Blvd, Savannah, USA). To ensure consistency, the coronal level of the root canal filling was standardized to be precisely 1 mm below the canal orifices. After root canal filling, any residual sealer that remained in the pulp chamber was carefully removed using a cotton pellet soaked in 70% alcohol, which aids in promoting a clean and dry environment conducive to coronal restorations. Moreover, to eliminate the potential residual effect of NaOCl (oxygen by-products) on subsequent polymerization of the adhesives and resin composite, access cavities were rinsed with an antioxidant agent (10% sodium ascorbate for 2 min), followed by water rinsing. Lastly, the quality of the root canal filling was meticulously assessed using conventional periapical radiographs, allowing for visual confirmation of the filling density and the proper apical seal. This step was crucial in verifying that the root canal filling met the necessary standards for effective root canal treatment.

The access cavity was meticulously sealed with a bulk-fill flowable resin composite (Tetric N-Flow Bulk Fill; Ivoclar Vivadent, Zurich, Switzerland) after applying the adhesive per the manufacturer’s instructions. This material was applied to effectively fill the majority of the access cavity, strategically leaving a 2 mm space to accommodate the final restoration with resin composite (Tetric N-Ceram, also from Ivoclar Vivadent). To ensure optimal curing of the resin composite, a high-performance light-emitting diode (LED) curing unit (the Blue Phase; Ivoclar Vivadent, Zurich, Switzerland) was utilized. This unit delivered a powerful light intensity of 1200 mW/cm² within a wavelength range of 400–515 nm, essential for achieving thorough polymerization. To maximize the effectiveness of the light-curing process, the curing tip was maintained in close and intimate contact with the surface of the tooth and restoration, minimizing potential light dispersion and enhancing curing efficacy. Furthermore, to guarantee consistent and reliable light output throughout the procedure, the Aphrodite LED radiometer CM-2500 (Motion Medical Supplies & Equipment Corporation, Sanzhong, Taiwan) was employed. This device enabled the precise standardization of light output, ensuring that the polymerization process met the required clinical standards and delivered optimal results for the restoration.

### Checking for the incidence of cracks and/or fractures

Following the completion of root canal procedures and subsequent coronal restoration, the samples underwent meticulous examination using a DOM at 20× magnification. This analysis aimed to identify any microscopic cracks or fractures that could potentially compromise the reliability of the mechanical testing results. After this critical evaluation, the samples were carefully stored in distilled water for 48 h, maintained at a controlled temperature of 37 °C and 100% humidity. This storage condition was deliberately chosen to ensure the complete setting of the sealer and to allow for thorough polymerization of the resin composite before submitting to dynamic thermo-mechanical loading, thereby ensuring that the material properties were fully developed and accurately reflective of clinical conditions.

### Dynamic thermomechanical fatigue (artificial aging)

Mechanical aging through cyclic loading was executed using advanced programmable logic-controlled equipment. The process utilized a newly developed four-station multimodal ROBOTA chewing simulator, engineered to simulate oral conditions with precision. This innovative simulator features an integrated thermo-cycling protocol and operates using a high-performance servo motor (Model ACH-09075DC-T, AD-TECH TECHNOLOG, Berlin, Germany), renowned for its reliability and efficiency.

The ROBOTA chewing simulator is designed with four independent chambers, each capable of mimicking vertical and horizontal movements concurrently, thereby replicating the complex dynamics of human chewing under thermo-dynamic conditions. Each chamber is equipped with an upper Jacob’s chuck, acting as a hardened steel stylus antagonist holder, which can be securely tightened with a screw to ensure stability during the simulation. The lower section of each chamber is constructed from Teflon, serving as the sample holder and allowing for easy removal and replacement of test samples without damaging them.

During the simulation process, a weight of 5 kg was applied to simulate a chewing force of approximately 49 N, reflecting real-life physiological conditions encountered within the oral cavity. The following operational settings were meticulously configured for the chewing simulation: a cycle frequency of 1.6 Hz, which corresponds to a realistic chewing rhythm; a forward rising speed of 90 mm/s to imitate the upward motion during mastication; a descending speed of 40 mm/s that reflects the controlled downward force; and lateral movement of 1 mm to accommodate the transverse motions of chewing. Additionally, vertical movement was set to rise 3 mm to enhance the simulation of the dynamic forces.

To comprehensively mimic six months of intraoral aging, the samples were subjected to a total of 75,000 cycles of mechanical loading. This rigorous regimen was supplemented with 5,000 thermal cycles, wherein temperatures fluctuated between 5˚C and 55˚C, thereby simulating the thermal stresses experienced in the mouth [21]. Each thermal cycle included a dwell period of 25 s, allowing the samples to stabilize at each temperature before undergoing the next phase of loading. This detailed approach ensured that the aging process closely mirrored actual clinical conditions, providing valuable insights into mechanical performance over time.

### Static fracture test

The static fracture test was conducted using a computer-controlled universal testing machine (Model 3345; Instron Industrial Products, Norwood, MA, USA). Each sample was secured firmly to the lower stationary compartment of the testing machine to ensure stability during the procedure. This was performed by tightening screws on a specialized holder, which effectively prevented any rotational movement that could affect the results.

For the testing, the machine was equipped with a high-precision 5-kN load cell that allowed for accurate measurement of the force applied. Data acquisition was facilitated through Bluehill Lite software (Instron), capturing load and deflection metrics at a frequency of 10 Hz.

**Fig. 1:**
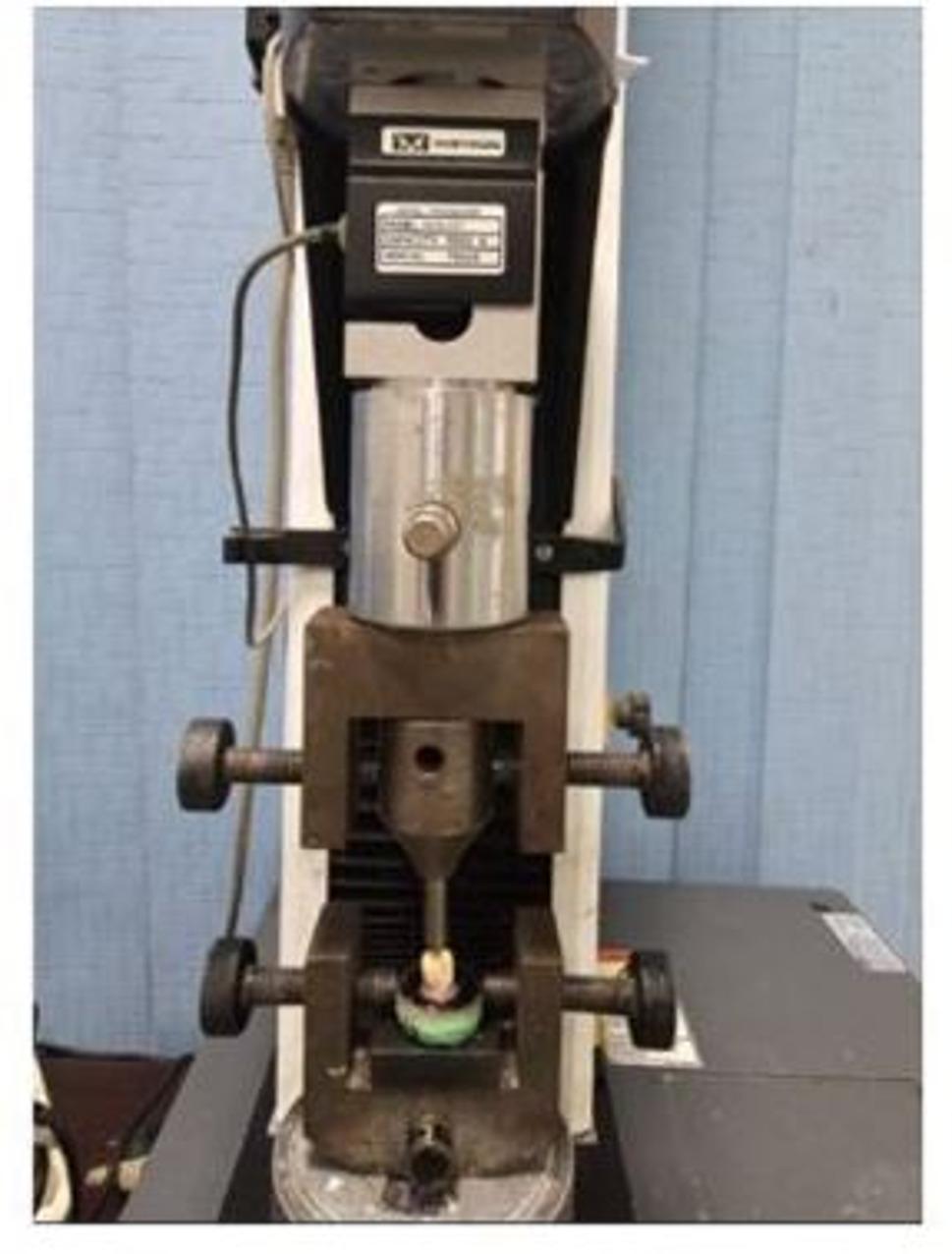
A representative image shows a specimen mounted on the universal testing machine during fracture resistance assessment. The tooth was embedded in acrylic resin, extending up to 2 mm below the cementoenamel junction to accurately simulate the alveolar bone, while a layer of light-body silicone was employed to replicate the periodontal ligament. A compressive load was applied occlusally utilizing a hemispherical metallic tip with a diameter of 3.8 mm, at a crosshead speed of 1 mm/min

A stainless-steel loading rod, featuring a hemispherical tip with a diameter of 3.8 mm, was affixed to the upper movable crosshead of the machine. The positioning of the rod was critical; it was carefully aligned to contact the cuspal inclines on the occlusal surface of the tooth. This ensured that the load was applied in optimal alignment along the long axis of the tooth, thereby mimicking the natural forces encountered during mastication (Fig. 1).

The crosshead was set to travel at a constant speed of 1.0 mm/min. As the compressive load was progressively applied, the specimens were monitored until failure occurred. Failure was defined as the moment an audible cracking sound was heard, accompanied by a noticeable and abrupt drop in the recorded load–deflection trace. At this point, the maximum load sustained until fracture incidence, measured in N, was meticulously recorded for each specimen.

Following the test, a qualitative assessment of the fracture pattern was conducted through visual inspection. The evaluator classified the fractures based on their observed characteristics.

### Failure pattern analysis

Following the completion of the fracture-resistance tests, all specimens from the various groups underwent a detailed examination using a USB digital microscope (U500× Digital Microscope, Guangdong, China) at a magnification of 35×. High-resolution images were captured and subsequently transferred to an IBM^®^ personal computer equipped with ImageJ software (v1.43U; National Institutes of Health, Bethesda, MD, USA) for further documentation and thorough measurement of the fracture lines.

**Fig. 2:**
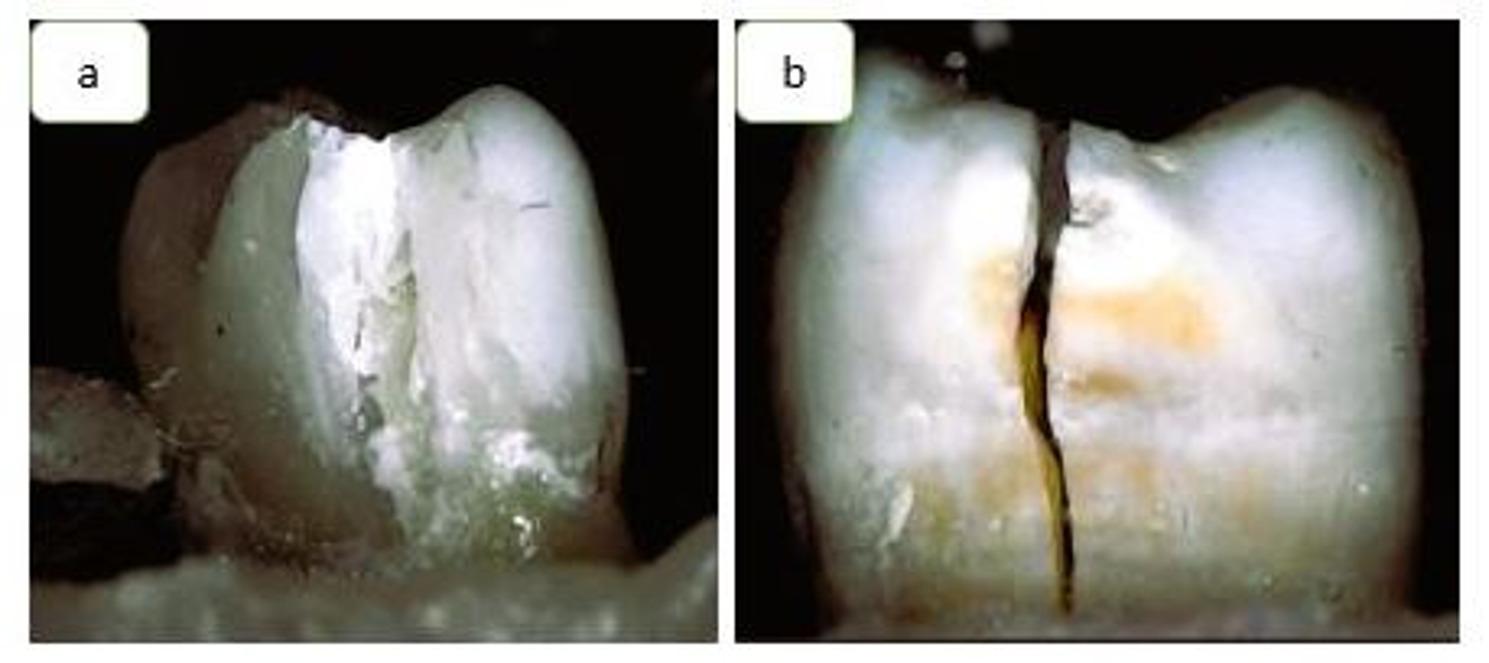
Representative images of failure modes: Favorable failure (**a**) and unfavorable failure (**b**)

Failure modes were systematically classified according to the location of the fracture line relative to the CEJ [22]. The classification was as follows (Fig. 2):

- Reparable (favorable): These fractures occurred above the CEJ, preserving enough tooth structure to allow for restorability.
- Catastrophic (unfavorable): In contrast, these fractures extended below the CEJ or involved the root in such a manner that the tooth could not be restored.

### Strategies to control confounding factors

In pursuit of valid, reliable outcomes and reproducibility of methodology, this laboratory investigation was executed under rigorously controlled conditions. The following strategies were employed:

- Sample Standardization:

The same tooth type was selected. Samples were meticulously matched for dimensions, anatomy, and age and evenly distributed across the various study groups. Prior to experimental procedures, all samples underwent cleaning and disinfection and were stored under consistent conditions regarding solutions, time, and temperature.

- Operator Experience and Skills:

All root canal and restorative procedures were performed by a single, experienced operator (M.H.A.I.) in a single setting under a controlled environment utilizing a DOM at 16× magnification to ensure methodological standardization. Operator blinding was not feasible due to the differences in irrigation protocols; however, a blinded evaluator (M.T.) conducted all fracture analyses to eliminate bias and maintain evaluation integrity. This rigorous approach upheld objectivity, ensuring unbiased assessment and integrity of the results for a clearer understanding of the findings’ implications. Statistical analyses were performed by a highly experienced specialist with three decades of data analysis expertise, who had no role in the current investigation, thereby providing an unbiased interpretation of the data and enhancing the study’s credibility.

- Standardization of Root Canal Instrumentation, Filling, and Restorative Procedures:

All samples underwent identical access cavity designs, root canal instrumentation, filling, and coronal restorations, with variations solely in the irrigation protocols to isolate their impact on the fracture resistance. The chemical solutions used in the present study were obtained from commercial stocks. Careful attention was given to the preparation of these solutions to maintain their integrity.

For accurate and consistent measurements, the pH levels of the solutions were quantified using a calibrated digital pH meter (HI 2210, Hanna Instruments Inc., Woonsocket, RI, USA), following the manufacturer’s instructions. Each measurement was recorded for each batch. The pH meter underwent routine calibration and testing to confirm its accuracy, thereby guaranteeing the reliability of the readings obtained.

To ensure precise concentration levels, the iodometric titration method was utilized, which is particularly effective for accurately quantifying the chemical composition of the solutions. This meticulous process involved a series of controlled titrations, enabling the verification of the exact concentrations required for the experimental procedures. Moreover, all solutions were used at room temperature. This attention to detail underscored the importance of precise conditions in experimental settings, ultimately contributing to the reproducibility and reliability of the results obtained.

While CSI was administered using the same needle design and gauge across the different samples, an identical tip design and size were utilized for ultrasonic irrigation. Additionally, total volumes and irrigation durations were consistently calibrated for each specific irrigation protocol. The penetration depths of the needle and ultrasonic tip were standardized to be 1 mm and 3 mm shorter than the working length, respectively, by positioning silicone stoppers at a fixed reference point. The instrumentation was performed using the same engine-driven file system and standardized apical size (0.40 mm), taper (0.04), timing, and frequency of file movements. Root canals were filled utilizing the same root canal filling technique and materials, and all access cavities were sealed uniformly. Before submitting to equivalent artificial aging conditions, all teeth were stored in the same storage solutions (distilled water) at standardized temperature and humidity for the same duration and assessed for evidence of cracks and/or fractures. This comprehensive standardization across operational parameters allowed for precise comparison of study outcomes.

- Environmental Conditions:

To ensure the reproducibility and reliability of laboratory studies, controlling environmental variables such as temperature and humidity is critical. Accordingly, the following methodologies were implemented:

1. Climate-Controlled Laboratory Environment:

A temperature-regulated laboratory was utilized, maintaining a consistent temperature of 23 °C ± 1 °C, per the ISO guidelines. Humidity levels were stabilized through the use of both humidifiers and dehumidifiers.

2. Environmental Chamber Utilization:

An environmental chamber was employed to provide precise control over both temperature and humidity parameters, enhancing the consistency of experimental conditions.

3. Continuous Ambient Monitoring:

Digital thermometers and hygrometers provided continuous monitoring of ambient conditions. Data was systematically recorded to ensure stability and to promptly identify any deviations.

4. Standardized Testing Protocols:

All samples were subjected to testing on the same day to mitigate variability. Furthermore, samples, instruments, materials, and testing apparatus were acclimatized in the same controlled environment to ensure thermal equilibrium prior to experimentation.

### Statistical analysis

Data analysis was performed utilizing R statistical analysis software (version 4.1.3 for Windows, Bell Laboratories, Murray Hill, NJ, USA). Normality of the data distribution was assessed using the Shapiro–Wilk test. As the data met the assumption of normal distribution, Levene’s test was employed to verify the homogeneity of variances. Given that the data revealed unequal variances among the groups, a Welch’s analysis of variance (ANOVA) was conducted to compare the mean maximum load to fracture across the different groups. The Games-Howell post hoc test was used for multiple pairwise comparisons. Results are reported as mean ± standard deviation (SD), with a 95% CI calculated to evaluate the precision of the estimates. Additionally, failure modes were examined among groups using the Chi-square (χ²) test. A significance level of *p* < 0.05 was set for all statistical analyses.

It is important to highlight that equivalence testing, such as the use of two one-sided tests, was not conducted in this study. The primary focus of the research was to identify and measure differences between the groups being analyzed, rather than to confirm equivalence. Furthermore, there were no predefined equivalence margins established prior to conducting the study. This design choice reflects the intention to explore differences in outcomes rather than to ascertain whether the groups fall within a specified range of similarity.

## Results

### Fracture resistance assessment

**Fig. 3:**
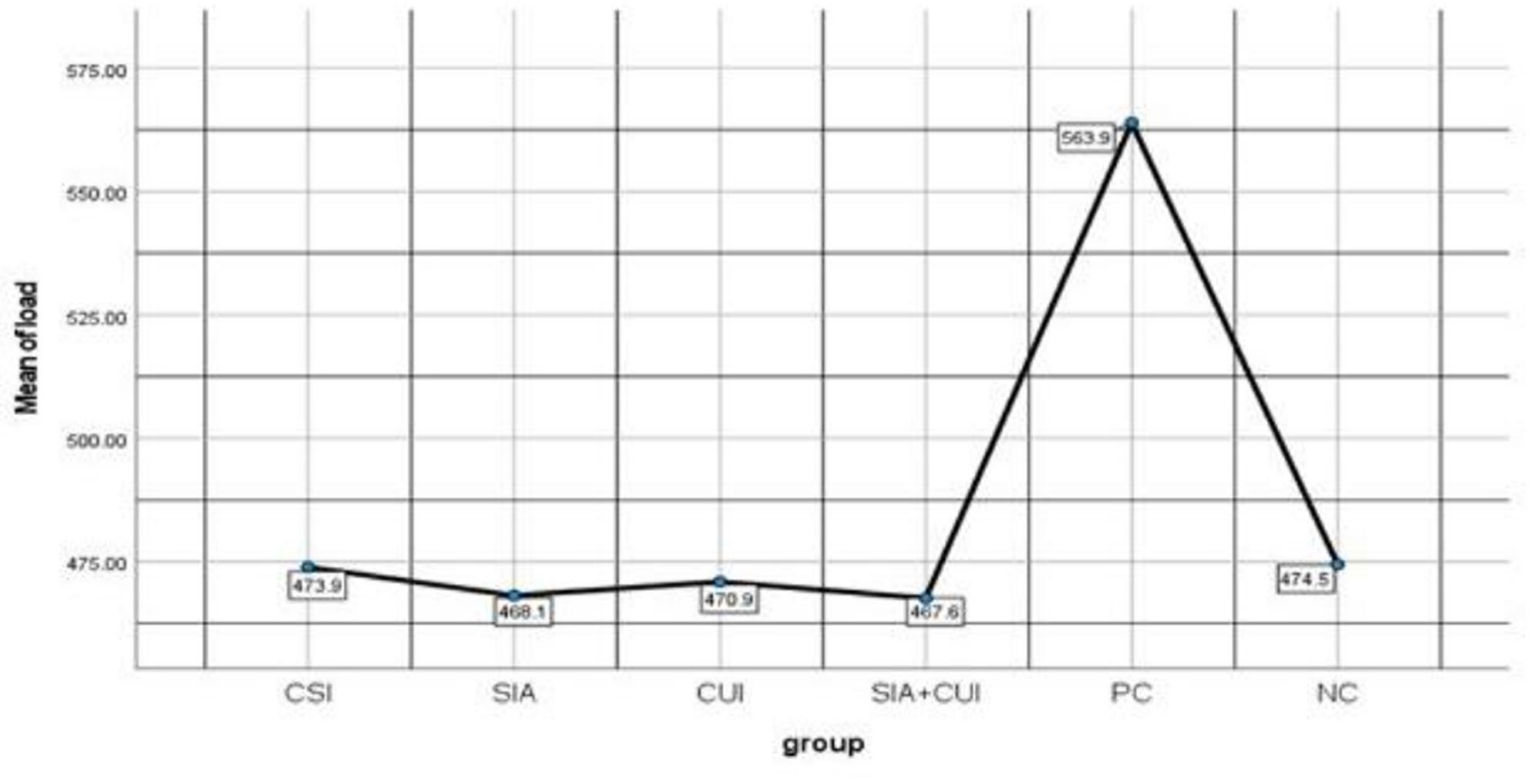
Line chart showing the maximum mean load to failure for each study group

**Fig. 4:**
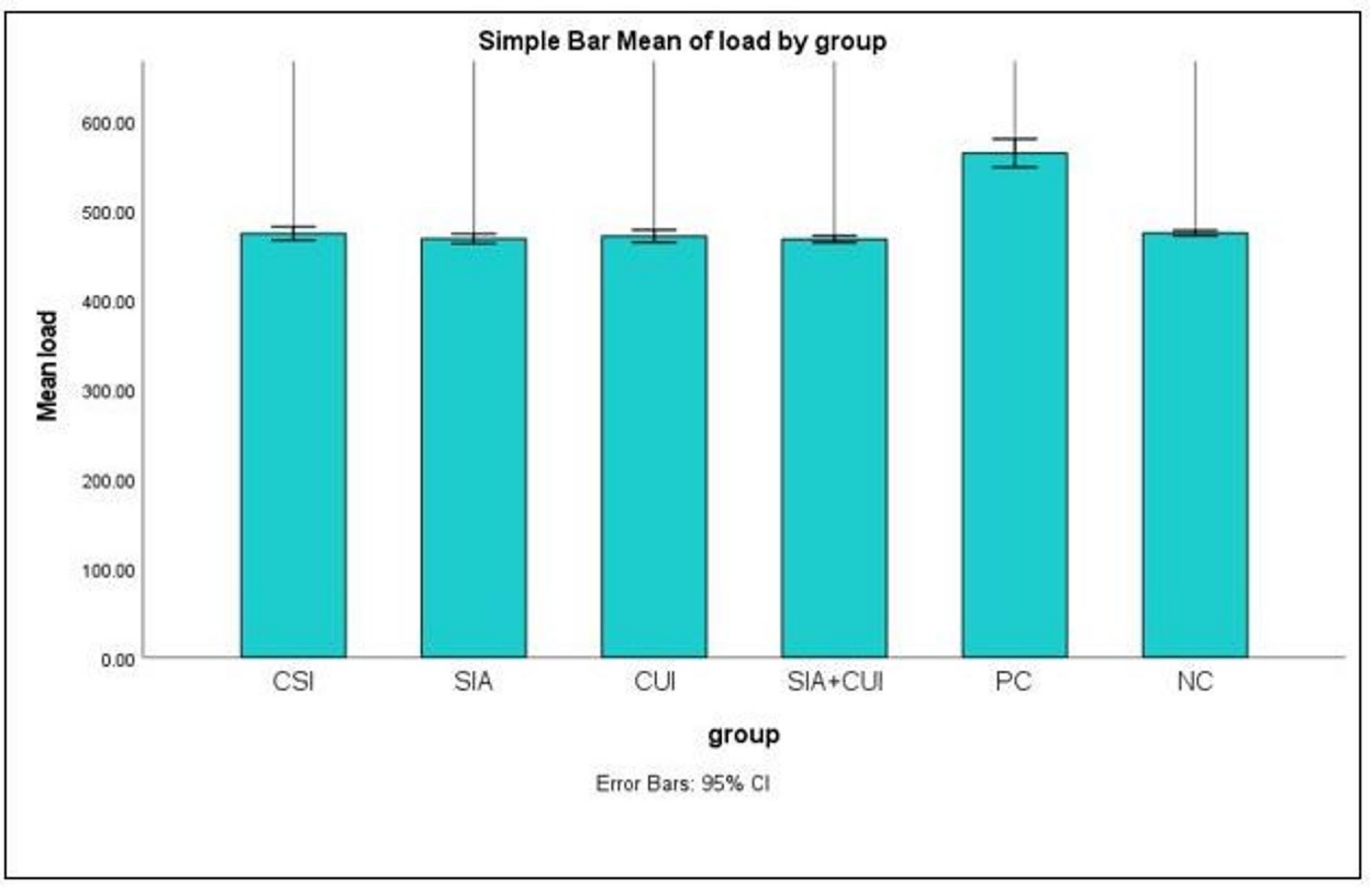
Fracture resistance trends plotted with error bars

Table 1 presents the means and standard deviations (SD) of the maximum load to failure for each study group. The PC group exhibited the highest mean fracture resistance (563.92 ± 28.52 N; CI: 548.13–579.71), followed by the NC group (474.45 ± 5.62 N; CI: 471.34–477.56), the CSI group (473.90 ± 13.94 N; CI: 466.18–481.62), the CUI group (470.94 ± 12.5 N; CI: 464.02–477.87), the SIA group (468.15 ± 9.68 N; CI: 462.79–473.51), and the SIA + CUI group (467.61 ± 6.74 N; CI: 463.88–471.35), respectively (Fig. 3). The fracture resistance trends plotted with error bars are shown in Fig. 4.

**Table 1:** The means and standard deviations (SD) of the maximum load to failure for each study group

Groups	*n*	Means ± Standard deviations.	95% Confidence Intervals		Minimum	Maximum	*p* value
			Lower Bound	Upper Bound			
CSI	15	473.90 ± 13.94 N^a^	466.18	481.62	450.10 *N*	492.40 *N*	*p* < 0.001
SIA	15	468.15 ± 9.68 N^a^	462.79	473.51	446.50 *N*	481.70 *N*	
CUI	15	470.94 ± 12.5 N^a^	464.02	477.87	451.30 *N*	490.60 *N*	
SIA + CUI	15	467.61 ± 6.74 N^a^	463.88	471.35	457.50 *N*	477.40 *N*	
PC	15	563.92 ± 28.52 N^b^	548.13	579.71	520.77 *N*	611.70 *N*	
NC	15	474.45 ± 5.62 N^a^	471.34	477.56	463.70 *N*	482.20 *N*	

Different superscript letters indicate statistically significant differences (*p* < 0.05)

*CSI* Conventional syringe irrigation, *SIA *Stepwise intraoperative activation, *CUI *Conventional ultrasonic irrigation, *PC *Positive control, *NC *Negative control, *n* Number of samples, *N* Newton

The PC group demonstrated significantly greater fracture resistance than all experimental and NC groups (*p* < 0.001). Importantly, no significant differences were identified among the other study groups, including CSI, CUI, SIA, SIA + CUI, and NC (*p* > 0.05).

The CIs for the mean fracture resistance values were relatively narrow, indicating a high level of precision in the estimates and reinforcing the validity of the results obtained.

### Failure mode analysis

Table 2 outlines the distribution of failure modes observed across groups. Catastrophic fractures were predominantly noted in the experimental and NC groups, as follows: CSI (60.0%), CUI (60.0%), SIA (73.3%), SIA + CUI (80.0%), and NC (53.3%). No significant differences were found among these groups (*p* = 0.06). Conversely, the majority of specimens in the PC group exhibited repairable fractures (93.3%), which represented a statistically significant difference compared to the other study groups (*p* = 0.02).

**Table 2:** Failure modes distribution across groups

Group	Catastrophic (%)	Repairable (%)
CSI	60.0^a^	40.0^a^
SIA	73.3^a^	26.7^a^
CUI	60.0^a^	40.0^a^
SIA + CUI	80.0^a^	20.0^a^
PC	6.7^b^	93.3^b^
NC	53.3^a^	46.7^a^

Different superscript letters indicate statistically significant differences (*p* < 0.05)

*CSI* Conventional syringe irrigation, *SIA *Stepwise intraoperative activation, *CUI *Conventional ultrasonic irrigation, *PC *Positive control, *NC *Negative control

The key findings of the present study indicated that while the various irrigation protocols did not result in statistically notable differences in fracture resistance among the experimental groups, a progressive decline in load-bearing capacity was associated with a higher tendency for catastrophic failure, particularly when compared to intact teeth.

While the analysis revealed no statistically significant differences in fracture resistance across the various experimental irrigation protocols, it is crucial to emphasize that these results should not be misconstrued as proof of equivalence among the groups involved, particularly given the inherent variability in the mechanical properties of dentin in real-world scenarios and the limitations of the current laboratory investigation. Therefore, the absence of significant differences in the present experiment warrants caution in generalizing these findings to practical applications.

## Discussion

The present laboratory investigation aimed to explore the effects of repeated activation of full-strength NaOCl on the fracture resistance of root canal-treated teeth. This study specifically examined the influence of larger volumes and extended exposure times to full-concentration NaOCl through CUI and SIA, both when used independently and in combination with CUI, compared to CSI.

Frequent and prolonged application of full-strength NaOCl has the potential to impact the properties of dentin. This influence might be attributed to its strong proteolytic activity on the organic matrix, particularly the type I collagen. NaOCl has the ability to oxidize and degrade collagen fibers, which are a crucial component of the dentin structure. This oxidative degradation can lead to a reduction in the mechanical strength of the dentin, ultimately diminishing its fracture resistance. Therefore, careful consideration should be given to the concentration, volume, and duration of NaOCl exposure to mitigate any adverse effects on the structural integrity of dentin.

However, the findings of this study revealed that all experimental groups—including CSI, SIA, CUI, and the combination of SIA with CUI—exhibited significant reductions in the fracture resistance of root canal-treated teeth. Notably, the failures observed in these groups tended to be more catastrophic. However, despite the variations in irrigation methods, particularly the increased volumes and prolonged exposure times to full-strength NaOCl employed in the CUI, SIA, and SIA + CUI groups, there were no statistically significant differences in fracture resistance or failure patterns among all the tested groups. Thus, the tested hypothesis was ultimately rejected.

The present results indicated that the mechanical integrity of root canal-treated teeth may be more profoundly influenced by the loss of tooth structure during the access cavity preparation and the root canal preparation process itself, rather than by the specific irrigation techniques applied. This observation was evidenced by the absence of statistically significant differences among the experimental and NC groups in terms of fracture strength and failure patterns, suggesting that the structural compromises inherent to the tooth preparation phases overshadowed any potential effects from the various irrigation methods. The rising occurrence of catastrophic fractures among groups with diminished fracture resistance may indicate a decline in the structural integrity of root canal-treated teeth. This deterioration can result from altered stress distribution within these teeth, which could compromise their ability to withstand mechanical forces. As a consequence, these teeth are more susceptible to unstable crack propagation when subjected to compressive loading.

Moreover, it is important to note that the application of irrigating solutions like NaOCl is typically fractionated rather than delivered as a continuous static soak. This practice is crucial in mitigating adverse effects on dentin’s structural integrity, which can deteriorate due to prolonged exposure to chemical agents, such as NaOCl. Although these findings corroborated previous investigations [23–26], they diverged from earlier studies that have reported significant reductions in fracture strength with increased volume and exposure time to 5.25% NaOCl [27, 28].

While the different activation protocols implemented—involving larger volumes and extended exposure times of full-strength NaOCl—did not influence fracture strength relative to CSI, previous research has indicated that CUI can improve fracture resistance when compared to CSI [15]. Conversely, another study evaluating CUI against a no-CUI final irrigation has found no significant difference in fracture resistance between the two methodologies [16], thereby echoing the current study’s findings that activation did not confer strength advantages over non-activated protocols. Additionally, findings from prior research utilizing bovine incisors have partially supported our observations, demonstrating that protocols employing heated NaOCl or PUI yielded lower fracture resistance compared to distilled water controls [17]. However, when comparing different active irrigation techniques, the findings were less consistent, indicating that the impacts of various methodologies can be context-dependent.

The discrepancies between our results and those reported in the existing literature can be attributed to several potential factors. First, while the volume and duration of NaOCl contact potentially play a role in dentin strength, our activation cycles, although more intensive, may not have exceeded the critical thresholds necessary to produce statistically significant differences among groups. Second, the thickness of the dentin serves as a crucial moderator; previous studies that have identified differences often utilized roots with thin residual dentin walls, which are inherently more vulnerable to weakening [15]. In contrast, the premolars used in the current study likely maintained sufficient dentin integrity to buffer against any weakening effects. Furthermore, variations in loading methodologies, rod geometry, embedding procedures, and crosshead speed may influence the sensitivity of the fracture tests applied; hence, some studies implementing extreme activation or stressors may have observed differences that our protocol did not detect. Finally, factors such as sample size, type, and inherent anatomical variability may also contribute to these discrepancies.

In conclusion, while these comparisons have indicated that full-strength NaOCl and activation cycles could enhance the exposure of dentin to potentially weakening chemical effects, under experimental conditions typical for premolars that retain significant structural dimensions, the differences in activation methods did not consistently translate into clinically or statistically meaningful differences in fracture resistance. Future research should aim to comprehensively report relevant parameters, including the number of activation cycles, total activation time with full-strength NaOCl, irrigation volumes, and dentin thickness, to better establish the thresholds at which activation begins to impair structural integrity. Moreover, it is worth noting that there are no prior studies that assessed the influence of SIA, either alone or in conjunction with CUI. Thus, further studies are required to validate the present data.

The clinical significance of these findings emphasizes the need to balance the antimicrobial effectiveness with the preservation of the tooth’s structural integrity. Based on the current results, clinicians might consider using SIA, either alone or alongside CUI, especially when enhanced disinfection is necessary. Importantly, within the parameters tested in this study, these irrigation methods—up to a maximum of 10 cycles of full-strength NaOCl activation—are unlikely to compromise the tooth’s mechanical strength beyond what is seen with standard irrigation techniques. However, the notable decrease in strength compared to intact teeth highlights the importance of carefully preserving remaining tooth structure and employing suitable post-treatment restorations to reduce the risk of vertical root fracture in patients. Understanding the biomechanical effects of root canal treatment is essential, as it can guide better strategies to improve the longevity and stability of these teeth under daily stressors.

To ensure valid and reliable results, this study sought to address several confounding factors that are often neglected in many laboratory investigations, particularly those testing fracture resistance. These factors included adherence to stringent criteria for sample selection, careful management of extraction time and technique, consideration of tooth age, optimal storage conditions, and the application of thermo-mechanical cycling to simulate real-life stresses experienced by teeth. By adopting this comprehensive approach, the study aimed to provide a highly controlled experimental setting, thereby translating the results into practical and meaningful clinical applications.

Maxillary premolars, particularly the second maxillary premolars, were chosen for this investigation due to their heightened susceptibility to fractures stemming from their reduced crown volumes and unfavorable crown-to-root proportions [29]. The selection process involved collecting freshly extracted teeth, where extractions were executed with minimal trauma to preserve their structural integrity.

Furthermore, the consideration of tooth age is often overlooked in ex vivo studies despite its significant implications. The literature has indicated that the dentin in younger patients (ages 17–30) demonstrates greater fatigue strength than that in older individuals (ages 50–80) [30]. In light of this information, this experiment was conducted using intact teeth sourced from healthy adult patients within a comparable age range of 20–30 years. This deliberate choice aimed to ensure uniformity in dentin characteristics and reduce the likelihood of any pathological or physiological alterations that could affect the results.

Storage conditions, specifically regarding duration and media, also represent critical factors that can significantly impact dentin properties and the overall structural integrity of teeth [31]. According to the technical specification 11–405 in the dental materials field, the most substantial alterations in dentin properties typically occur within the initial days or weeks after extraction. Therefore, teeth are ideally tested one month following extraction, but not beyond six months. In this research, all teeth were freshly extracted and carefully stored in the same preservation medium for a duration not exceeding one month prior to testing. By adhering to this short-term storage protocol, the use of thymol solution would be an effective method for preserving the mechanical characteristics of the recently extracted teeth [32].

The concept of thermo-mechanical fatigue was incorporated into the study design to replicate the natural stresses that teeth endure during oral function over time. This fatigue can lead to collagen degradation and negatively impact the fracture resistance of the tooth structure [33]. Thus, the implementation of thermo-mechanical cycling in this investigation was essential to accurately mimic clinical scenarios, further reinforcing the validity of the findings.

In the present study, access cavities were prepared instead of removing the tooth’s crown as part of the experimental setup. This decision was made to better simulate clinical situations, where the design and preparation of access cavities can significantly influence subsequent root canal procedures, including effective root canal irrigation—the main focus of the present study [34]. The current research specifically emphasized the design of these access cavities, opting for traditional designs that could facilitate efficient root canal and restorative procedures while minimizing the risk of procedural errors commonly associated with minimally invasive access cavities [34].

The optimal size for root canal preparation remains a topic of debate, with evidence leaning towards the advantages of larger apical preparations [35]. Larger sizes facilitate the mechanical disinfection provided by instruments and offer adequate space for deep, passive insertion of irrigating needles and ultrasonic inserts. This also results in increased volumes and exposure times to irrigants, significantly enhancing irrigation effectiveness, particularly in the apical region. Current literature has suggested that a minimum apical preparation size corresponding to file sizes of 30–35 is essential for effective cleaning and disinfection of the root canals [11]. Contrastingly, canal taper appears to have a limited impact on irrigation efficacy [36]. Some reports have demonstrated that enlarging root canals to size 45 in premolars could compromise the fracture resistance of the tooth [37]. Moreover, it has been noted that root canal preparation up to size 45/0.04 may not add significant improvement in root canal debridement compared to size 40/0.4 in premolar teeth [38]. Considering all these biological and mechanical factors, the selected single-rooted maxillary second premolars were prepared to size 40/0.04 to potentiate the clinical relevance of the present laboratory study.

Unlike several previous laboratory investigations, root canal filling and restorative procedures were performed in the present study, closely replicating a realistic clinical setting. To minimize procedural variability and mitigate confounding stresses from root canal filling techniques, the study employed the single-cone obturation method. Despite the reported non-significant differences, traditional techniques, such as lateral compaction and warm vertical condensation, can create wedging forces within the canal, potentially leading to cracks and reduced fracture resistance [39]. Conversely, the single-cone technique applies minimal mechanical stress and can achieve a satisfactory filling, especially when combined with contemporary sealers [39]. This choice allows for a more precise assessment of the impact of irrigation and activation protocols on the mechanical properties of root canal–treated teeth. Nonetheless, clinicians should be cautious about the appropriateness of this technique for each specific case, especially in the presence of intricate root canal anatomies, where the single-cone approach may not be the best option [40].

While NaOCl is renowned for its potent antimicrobial efficacy and unique ability to dissolve organic tissue, it has a significant limitation: it cannot effectively dissolve the inorganic components of the smear layer generated during canal instrumentation. To address this deficiency, using a chelating agent, specifically 17% EDTA, is advocated to enhance the removal of the smear layer. Not only does EDTA aid in removing this layer, but it also has the potential to improve the antibiofilm properties of NaOCl, albeit with limited antimicrobial activity itself [41]. This enhancement is achieved by facilitating the detachment of biofilm structures from the canal walls [41]. Consequently, while NaOCl is traditionally applied during mechanical file use, its application should ideally be complemented by EDTA as a final rinse at the conclusion of chemo-mechanical preparation [2]. The present study considered the severe consequences that can arise from combining these two irrigants [2], as well as the possible negative effects on the dentin structure if EDTA is used before NaOCl [42]. Therefore, the standardized protocol was employed, incorporating the sequential use of 10 mL of 5.25% NaOCl, followed by 10 mL of 17% EDTA, each for a duration of 2 min, with an intermediate rinse of saline solution to mitigate potential complications [43]. To counteract the persistent softening of canal walls by EDTA, a final flush of saline solution was administered [2].

The design of the irrigating needle can significantly influence irrigation efficacy [44]. In line with existing literature advocating for the use of side-vented, closed-end irrigating needles to mitigate the risk of unintended irrigant extrusion, especially with higher concentrations of NaOCl [11], this study employed a side-vented, closed-end needle. This choice was made despite the acknowledged superior efficiency of open-ended needles in effectively cleaning and disinfecting the apical region [44].

There are two forms of ultrasonic irrigation: intermittent and continuous passive ultrasonic agitation. It has been shown that repetitive oscillation leads to vigorous streaming, which significantly enhances cleaning effectiveness and results in more efficient biofilm removal compared to continuous activation of the same duration [45, 46]. Additionally, regularly replacing the irrigating solutions compensates for their depletion from the pulp chamber and their consumption in chemical reactions [7, 47]. Thus, the current study opted for intermittent ultrasonic activation to optimize fluid dynamics during irrigation. To maximize the effects of ultrasonic activation, the ultrasonic file or tip must be inserted passively into the root canals. This approach minimizes the risk of fracture and prevents cutting into the canal walls while generating stronger acoustic streaming and cavitation effects. This shift in methodology led to the terminology change from “active ultrasonic activation” to “passive ultrasonic irrigation.” Research has indicated that smaller activated inserts yield more powerful cavitation and acoustic streaming, thereby exerting greater shear stresses on the root canal walls and eliminating apical vapor lock [2, 11]. Consequently, an ultrasonic tip with a size of 20 and a 0.01 taper was utilized in this study.

There are two main approaches for testing fracture resistance: dynamic and static testing. Given that static fracture testing is the most widely accepted method due to its reliability and capacity to produce comparable results [48], it was chosen for this study. While dynamic testing could more accurately reflect clinical conditions, variations in its design have been shown to affect the correlation of results [48], leading to the decision to forgo this method in favor of the more standardized static approach.

The primary limitation of the present research lies in its ex vivo design. This study was conducted using extracted human premolars in a controlled laboratory setting, which may not fully mimic the intricate and dynamic conditions found in the oral cavity. Factors such as variations in functional loading, solution renewal rates, ambient temperature changes, and the unique anatomy of each canal can significantly impact the results and may lead to variability in clinical outcomes. Furthermore, the study lacked an assessment of the long-term degradation of dentin collagen and the potential structural alterations that may occur over time. This oversight significantly hampers the ability to accurately predict the cumulative effects of repeated full-strength NaOCl activation in a live biological system (in vivo). Understanding how these factors interact over prolonged periods is crucial for evaluating the overall efficacy and safety of irrigation protocols used in endodontic treatments. Without this critical long-term analysis, the clinical implications of repeated full-strength NaOCl activation and its impact on dentin integrity remain unclear. This study concentrated specifically on fracture resistance as a primary mechanical outcome. However, it is important to note that other critical properties of dentin, including microhardness and elastic modulus, were not evaluated within the scope of this research. These properties play a vital role in understanding the overall mechanical behavior and structural integrity of dentin. Therefore, future studies should aim to investigate these additional dentin characteristics in relation to different full-strength NaOCl activation protocols. By doing so, researchers and clinicians can gain a more comprehensive understanding of how these protocols influence the structural dynamics of dentin, ultimately contributing to improved clinical outcomes in dental practice. Moreover, intact teeth were selected for the present study, which is not the case for those indicated for root canal treatment. These concerns highlight the need to approach the findings with caution, as they may not be broadly applicable in a real-world clinical context.

Contrarily, the main strengths of the current study are its novelty and its substantial contribution to clinical practice. This investigation represents the first direct comparative analysis of SIA techniques, evaluating their effects both individually and in conjunction with the CUI of full-strength NaOCl on the fracture resistance of root canal-treated teeth. By examining these techniques in a comparative framework, this research can provide valuable insights that could significantly enhance the outcomes of root canal procedures in clinical settings.

To advance this research field, further studies should delve into the synergistic effects arising from the combination of such innovative irrigation activation methods alongside minimally invasive access or instrumentation techniques. Additionally, it would be beneficial to investigate a broader range of irrigants, including different concentrations, to evaluate their efficacy in promoting favorable outcomes. Future research should ideally involve larger, multicenter studies to bolster statistical power and reinforce the generalizability of the findings. Ultimately, prospective clinical research that connects these emerging irrigation strategies with long-term outcomes, such as tooth survival rates and the incidence of fracture, would provide invaluable insights. Such evidence would be essential for informing best practices and guiding clinicians in making evidence-based decisions that enhance patient care.

## Conclusion

Within the constraints of this ex vivo analysis, the repeated activation of full-strength NaOCl, whether through stepwise intraoperative activation or conventional ultrasonic irrigation methods, or their combination, did not considerably reduce the fracture strength of root canal-treated teeth relative to conventional syringe irrigation, despite the utilization of larger volumes and prolonged exposure times in the activated approaches. These results can provide significant clinical implications, highlighting that repeated activation of fresh full-concentration NaOCl, when executed following standard access and instrumentation protocols, can be used when enhanced canal debridement and disinfection are required, without further jeopardizing the mechanical integrity of root canal–treated teeth. Still, meticulous preservation of tooth structure remains critical in mitigating fracture risk during clinical interventions.

## Supplementary Information

Supplementary Material 1.

Supplementary Material 2.

## Data Availability

All data or materials generated or analyzed during this study are included in this article.

## Abbreviations

NaOCl: Sodium hypochlorite
SIA: Stepwise intraoperative activation
CUI: Conventional ultrasonic irrigation
CSI: Conventional syringe irrigation
CI: Confidence interval
PUI: Passive ultrasonic irrigation
PRILE: Preferred Reporting Items for Laboratory Studies in Endodontology
ANOVA: Analysis of variance
3D: Three-dimensional
CBCT: Cone-beam computed tomography
FOV: Field of view
DOM: Dental operating microscope
CEJ: Cementoenamel junction
ISO: International Organization for Standardization
EDTA: Ethylenediaminetetraacetic acid
PC: Positive control
NC: Negative control
Newton: N
LED: light-emitting diode
Hz: Hertz
^o^ C: Celsius
h: Hours
min: minutes
s: Seconds
SD: Standard Deviation
mm: Millimeter
nm: Nanometer
mL: Milliliter
Kg: Kilograms
