# Supplementary material for: Influence of repeated cycles of full-strength sodium hypochlorite activation on the mechanical behavior of root canal-treated teeth: a laboratory study
Source: BMC Oral Health. 2026 Apr 1;26:635. doi: 10.1186/s12903-026-08082-3 (PMC13063722; doi:10.1186/s12903-026-08082-3)
Supplement: Supplementary file 2 — Supplementary Material 2. [file 12903_2026_8082_MOESM2_ESM.docx]

**Supplementary Figure** **1.** The Preferred Reporting Items for Laboratory Studies in Endodontology (PRILE 2021)* flowchart, showing the study design, sample allocations, interventions, and outcome evaluation of the current investigation.

***From Nagendrababu V, Murray PE, Ordinola-Zapata R, Peters OA, Rôças IN, Siqueira JF Jr, Priya E, Jayaraman J, Pulikkotil SJ, Camilleri J, Boutsioukis C, Rossi-Fedele G, Dummer PMH (2021) PRILE 2021 guidelines for reporting laboratory studies in Endodontology: a consensus-based development. *International Endodontic Journal* May 3. doi: 10.1111/iej.13542.** [**https://onlinelibrary.wiley.com/doi/abs/10.1111/iej.13542**](https://onlinelibrary.wiley.com/doi/abs/10.1111/iej.13542)**.**

**For further details visit: http://pride-endodonticguidelines.org/prile**
